# Simultaneous polarization filtering and wavefront shaping enabled by localized polarization-selective interference

**DOI:** 10.1038/s41598-020-71508-7

**Published:** 2020-09-02

**Authors:** Jixiang Cai, Fei Zhang, Ming Zhang, Yi Ou, Honglin Yu

**Affiliations:** 1 Key Laboratory of Optoelectronic Technology and System, Ministry of Education, Chongqing University, Chongqing, 400030 China; 2 School of Information Science and Engineering, Hebei University of Science and Technology, Shijiazhuang, 050018 China

**Keywords:** Metamaterials, Metamaterials, Two-dimensional materials

## Abstract

The ability of simultaneous polarization filter and wavefront shaping is very important for many applications, especially for polarization imaging. However, traditional methods rely on complex combinations of bulky optical components, which not only hinder the miniaturization and integration but also reduce the efficiency and imaging quality. Metasurfaces have shown extraordinary electromagnetic properties to manipulate the amplitude, polarization, and wavefront. Unfortunately, multi-layer metasurfaces with complex fabrication are often required to realize complex functions. Here, a platform of monolayer all-dielectric metasurfaces is proposed to simultaneously achieve polarization filtering and wavefront shaping, based on the principle of local polarization-selective constructive or destructive interference. The transmission efficiency surpassing 0.75 and polarization extinction ratio exceeding 11.6 dB are achieved by the proposed metasurface at the wavelength of 10.6 μm. These results are comparable to those of multi-layer metasurfaces. Considering these good performances, this work may prove new ideas for the generation of complex optical field and find wide applications in polarization imaging.

## Introduction

Manipulating polarization and wavefront of light plays an important role in optical systems, which attracts great attention from researchers. In traditional optical systems, polarization is mainly controlled by anisotropic medium or the devices based on the Brewster effect. By tuning geometry and refractive index distribution, these methods can introduce anisotropy phase accumulations in the direction of transmission and then achieve wavefront manipulation^1–3^. Numerous optical components have been designed based on the aforementioned methods, such as wave plates^4^, polarizers^5^, phase retarders^6^, and various lenses^7,8^. However, simultaneous polarization and wavefront manipulation, commonly used in polarization imaging systems, are achieved by a complex combination of optical components^9,10^. Since different targets have unique polarization characteristics, polarization imaging has been widely applied in the fields of environmental science, medical diagnosis, astronomy detection, highlighting targets, and so forth^11^. However, traditional imaging systems require large volume and weight to achieve both wavefront shaping and polarization filtering, which is against the development trend of lightweight, integration, and planarization of modern optics^11,12^.

As two-dimensional artificially structured materials, metasurfaces have attracted tremendous attention owing to their unprecedented properties of optical manipulation^13–16^. Flexible amplitude, phase, and polarization manipulations can be achieved at the subwavelength scale using metasurfaces with subwavelength thickness^17–20^, providing a basis for the next-generation Engineering Optics 2.0^21^ . Many exotic phenomena and fascinating flat optical devices have been realized using metasurfaces, such as anomalous refraction^22,23^, optical holograms^24–26^, vortex generators^27–29^, and polarization converters^30–33^, among many others^34^. Simultaneous control of polarization and wavefront has been demonstrated by high-contrast dielectric elliptical nanoposts with high-efficiency^32^. To simultaneously realize polarization filtering and wavefront shaping, previous methods usually require to cascade multiple metasurfaces at the cost of complex fabrication and low efficiency^35–39^. By adjusting the orientation and duty cycle of the sub-wavelength metal–insulator–metal (MIM) gratings, both polarization and phase can be flexibly controlled, but the cost of the large pixel period is required to maintain the performance^36^. However, the large pixel period can not sufficiently sample the phase distribution, leading to low imaging resolution and efficiency^40,41^. It is still a great challenge to simultaneously achieve polarization filtering and wavefront shaping in the subwavelength scale, limiting the development of lightweight, planarized, and integrated optical systems, especially polarization imaging systems.

In this paper, a platform of monolayer all-dielectric metasurfaces is proposed to achieve simultaneous polarization filtering and wavefront shaping via the local polarization-selective interference of a supercell consisting of two pairs of nanofins. When illuminated by two orthogonal linear polarizations, each of them exhibits the unique resonant response due to the local constructive and destructive interference. Benefiting from lossless material and optimized nanofin geometry, high transmission efficiency (75.56%) and extinction ratio (11.6 dB) have been demonstrated. Without additional polarizer and complicated multilayer structure, the proposed metasurface could be further integrated with the spectral filter to realize multi-spectral polarization imaging, holding enormous potential applications.

## Results and discussion

**Figure 1:**
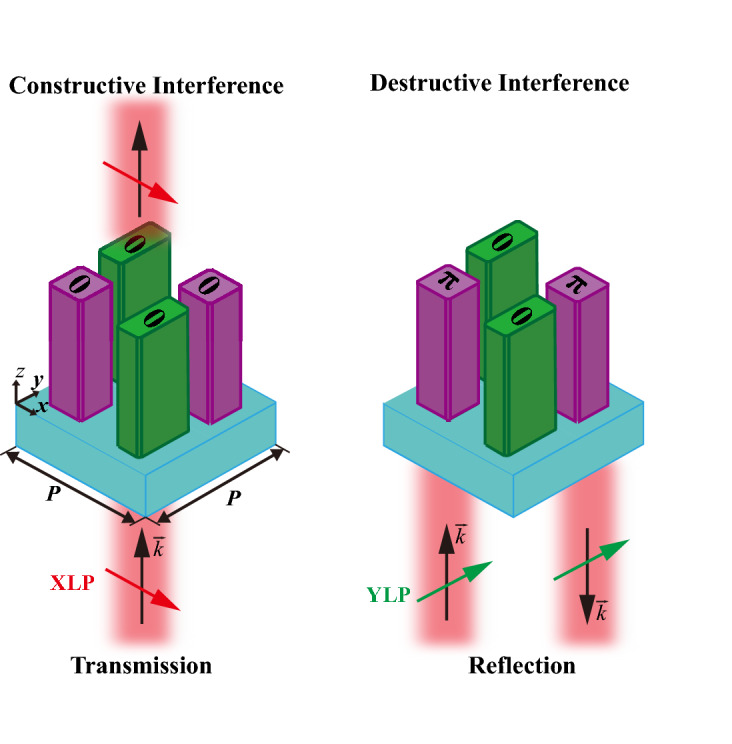
The schematic of local constructive or destructive interference ($\overrightarrow {k}$ is the wave vector and *P* is the period of supercell).

The principle of local constructive and destructive interference in the subwavelength range (*P* < λ) is shown in Fig. 1. Since adjacent nanofins provide none phase difference, the X-linear-polarized (XLP) incidence experiences local constructive interference, which can realize near 100% transmission in theory. However, the Y-linear-polarized (YLP) incidence undergoes phase difference of π and therefore is only reflected. As a result, localized polarization-selective interference is achieved for polarization filtering. To simultaneously achieve wavefront shaping, the phase of the transmitted light should cover 0–2π under the condition of constructive interference. Assuming an 8-level phase quantization, eight supercells with the phase interval of π/4 are required, that is, we need to optimize eight pairs of green and purple nanofins according to the following two conditions. First, the green and purple nanofins in a supercell have a phase difference of 0 for the XLP incidence but π for the YLP incidence. Second, the phase difference between adjacent green/purple nanofins is equal to π/4 for the XLP incidence but zero for the YLP incidence.

**Figure 2:**
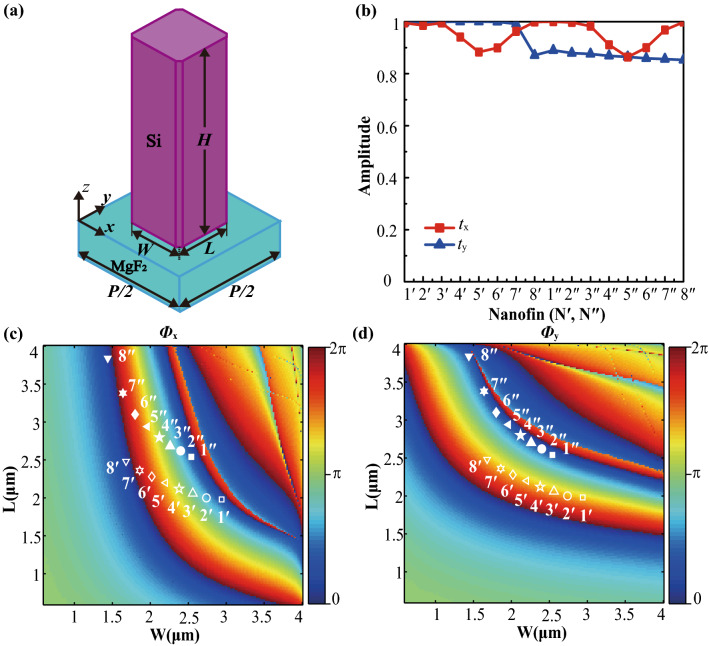
(**a**) 3D view of the subunit element. (**b**) Amplitudes of sixteen subunit elements, where *t*_x_ and *t*_y_ indicate the amplitudes for XLP and YLP incidence, respectively. (**c**,**d**) Phase shifts of sixteen subunit elements for (**c**) XLP and (**d**) YLP incidence.

Without losing the universality, the proposed method for simultaneous polarization filtering and wavefront shaping is elaborated aiming at a wavelength of 10.6 μm, which is the working wavelength of the CO_2_ laser. The subunit element, consisting of the magnesium fluoride (MgF_2_) substrate and 9.5 μm thick silicon (Si) nanopillar, is schematically illustrated in Fig. 2a. The refractive indices of MgF_2_ and Si are 1.17 and 3.42, respectively^42^. The designed eight green and eight purple nanofins, respectively named as 1′–8′ and 1″–8″, have the same height but different horizontal sizes. (Their geometrical parameters are listed in Supplementary Table S1.) The amplitude of transmission for sixteen nanofins is calculated by the CST Microwave Studio based on finite integration technology (FIT), as shown in Fig. 2b. The amplitude difference among green/purple nanofins is small, which creates a precondition for constructive or destructive interference.

**Figure 3:**
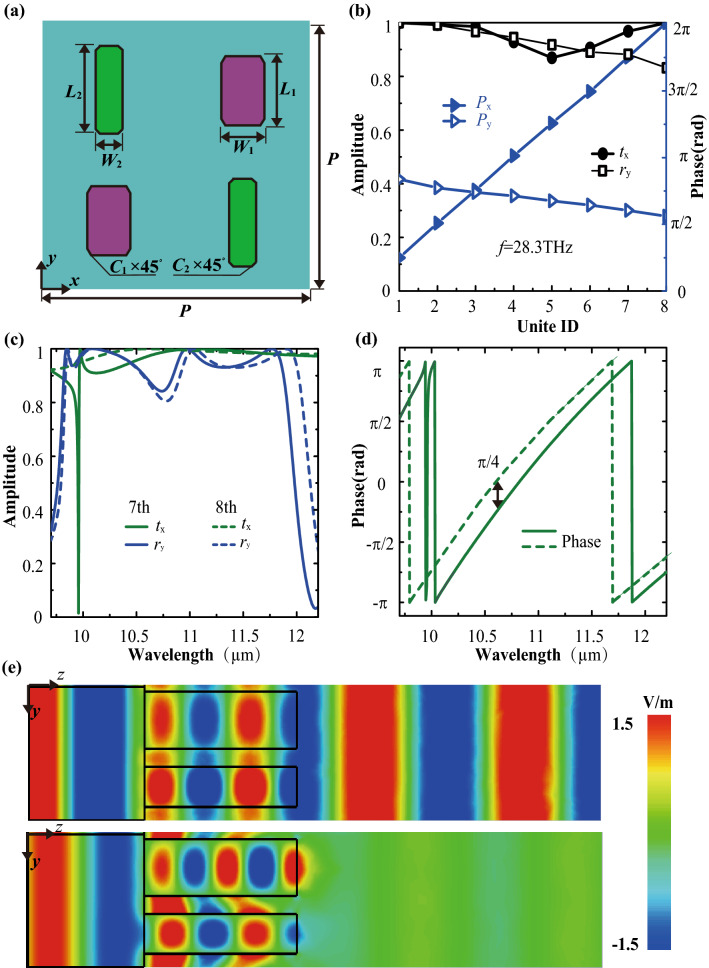
(**a**) Top view of a localized polarization-selective interference supercell. (**b**) Transmission (*t*_x_) and reflection (*r*_y_) amplitudes and corresponding phase shifts (*P*_x_, *P*_y_) of eight supercells for XLP and YLP incidence, respectively. (**c**) Amplitude and (**d**) phase spectra of the 7th and 8th supercells. (**e**) Electric field *E*_x_/*E*_y_ distributions of the 7th supercell under the illumination of XLP/YLP (top/bottom) light from the substrate side.

According to the aforementioned two conditions of designing nanofins, Fig. 2c shows the phase distribution (*Φ*_*x*_) of the nanofin as the function of its width and length at the XLP incidence. As can be seen, the phase shifts of designed eight green/purple nanofins cover 0–2π with an interval of ~ π/4, and the phase difference between green and purple nanofins with the same ID (e.g., 1′ and 1″) is approximately equal to zero, leading to constructive interference and arbitrary wavefront manipulation. Furthermore, the average amplitude of ~ 0.96 is supported by these sixteen subunit elements. Due to the rotational symmetry of the nanofins, the phase distribution (*Φ*_y_) for YLP incidence is a mirrored image of that of *Φ*_*x*_ in Fig. 2c flipped along the *W* = *L.* As shown in Fig. 2d, the phase difference between 1′ and 1″ maintains ~ π (others have the same characteristic) and eight green/purple nanofins have the same phase shift, resulting in destructive interference and specular reflection. As schematically shown in Fig. 3a, the supercell consists of two pairs of green and purple nanofins with the same ID, which can produce localized polarization-selective constructive or destructive interference. The period of the supercell is 8 μm. Figure 3b plots the transmission/reflection amplitude, and corresponding phase shifts of eight supercells for XLP/YLP incidence. Their transmission phase shifts can cover 0–2π for XLP incidence, but their reflection phase shifts almost the same for YLP incidence. Furthermore, the high average transmission amplitude of 0.96 is supported for XLP incidence, and the high average reflection amplitude of 0.92 is allowed for YLP excitation. These performances are affected by the dimension of supercells, thus red shift can be observed in both the amplitude and phase spectra with the height of supercells varying from 9.5 to 10.5 μm, as shown in Supplementary Fig. S1. Besides, the red/blue shift for the amplitude and phase spectra is negligible as the value of chamfers reduces/increases (Supplementary Fig. S2). As a result, these supercells can be regarded as subwavelength polarization filters with an ability of arbitrary wavefront manipulation.

For a further explanation, Fig. 3c illustrates the transmission and reflection amplitude spectra of the 7th and 8th supercells. Within the wavelength range from 10 to 11.8 μm, *t*_x_ and *r*_y_ of the 7th and 8th supercells exceed 0.91 and 0.86, respectively. Meanwhile, the phase difference between them maintains ~ π/4, as shown in Fig. 3d. The electric field distribution (*E*_*x*_) at XLP incidence is shown at the top of Fig. 3e. The phase difference between green and purple nanofins approximates 0, resulting in constructive interference and full transmission. While YLP incidence undergoes destructive interference being reflected, as shown in the bottom of Fig. 3e. Furthermore, if we judiciously design supercells to satisfy both conditions of constructive/destructive interference and phase combination (Assuming 8-level phase quantization are optimized to cover 0–2π for the XLP and YLP light. A total number of 8 × 8 supercells are needed, each of which realizes special phase shifts for two different polarized light incidence.), it is possible to simultaneously manipulate reflected and transmitted beam.

## The design of deflector and metalens

**Figure 4:**
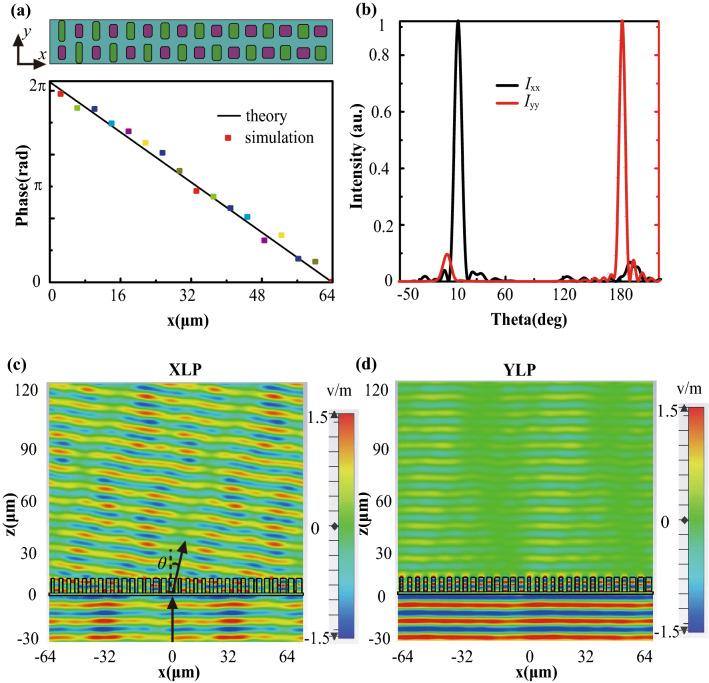
Simulated phase and electric field distribution of the deflector. (**a**) Top view of phase distribution in the *xoy* plane and the phase distribution along the *x*-direction in the *xoz* plane. (**b**) Far-field intensity distributions under the illumination of XLP (*I*_xx_) and YLP (*I*_yy_) lights. (**c**, **d**) The distribution of real (*E*_x_) and real (*E*_y_) on the *xoz* plane for XLP and YLP excitation.

Phase manipulation of the metasurface produces various optical effects. To verify the feasibility of the designed scheme, an efficient deflector capable of polarization filtering is designed based on generalized Snell’s law^43^ and the deflection angle is:

1 $$\theta = arcsin(\lambda /P)$$

where *P* is the period of the metasurface along the *x*-direction, and λ is the wavelength of the incident light. The top view of the deflector in the *xoy* plane is schematically illustrated in Fig. 4a. Gradient metasurface with a linear varied phase profile is realized, and the theoretical and simulated phase distribution of transmission shows a good agreement under XLP excitation. The intensity distribution of the far field along the phase is shown in Fig. 4b. The XLP incidence is almost completely transmitted with a refraction angle of ~ 9.53°, but the YLP incidence is directly reflected. Figure 4c,d shows the electric field distributions of the *x*-components and *y*-components when excited by XLP and YLP, respectively.

The minimum transmission efficiency, diffraction efficiency, and extinction ratio of the designed deflector within 10.2 to 11.6 μm exceed ~ 60%, ~ 60%, and ~ 12.92 dB, respectively, as shown in Supplementary Figure S3. The transmission/diffraction efficiency is defined as the ratio of transmitted power toward a specific angle to the total incident/transmitted power^44,45^. The extinction ratio is defined as ER = 10*log(*η*_x_/*η*_y_), where *η*_x_ and *η*_y_ indicate transmitted efficiencies of XLP and YLP illumination, respectively. It can be observed that the transmission efficiency, diffraction efficiency and extinction ratio reaches ~ 85.6%, ~ 92.24%, and ~ 19.3 dB at the wavelength of 10.6 μm, respectively. By an orderly arrangement of unit elements with gradient slopes in the phase spectra, the deflector can be designed to achieve maximum deflection angle ~ 26.2° (see Section S2 in Supplementary Information for details). Note that half-power beam-width (HPBW) of the deflector for 8-level phase quantization is ~ 4.1°, which can be easily decreased by increasing the size of the deflector. For example, the HPBW of ~ 0.8° can be realized with the size 640 μm, as shown in Supplementary Table S3. These results prove that the designed deflector can simultaneously achieve wavefront shaping and polarization filtering with high efficiency.

To further show the ability of polarization imaging by the proposed platform, a planar cylindrical metalens has been designed. The relationship between phase distribution and position coordinates is as follows^36^:

2 $$\varphi (x){ = }\frac{2\pi }{\lambda }(\sqrt {x^{2} + f^{2} } - f)$$

where *f* = 430 μm is the focal length of the cylindrical metalens.

**Figure 5:**
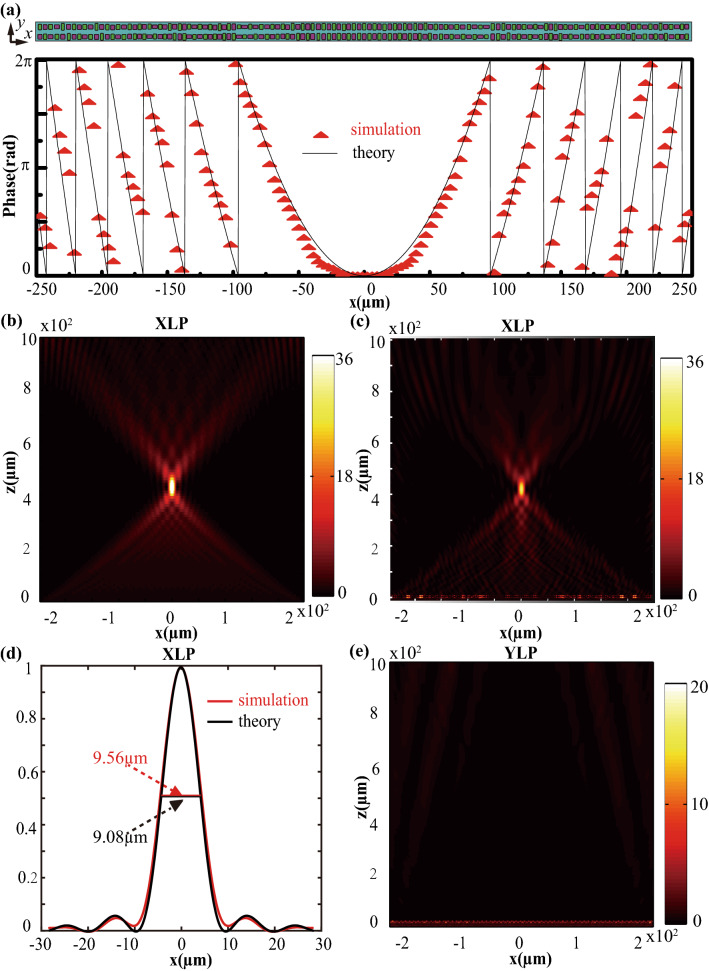
(**a**) Cross-sectional view of *xoy* plane composed of supercells and theoretical and simulated phase profile in the *x*-direction. (**b**,**c**) The theoretical and simulated cross-section intensity distributions of the planar lens along the *z*-axis for XLP incidence with λ = 10.6 μm. (**d**) The sectional intensity curves of simulation and theory calculations in the *z* = 430 μm plane. (**e**) Cross-section intensity distribution of the planar lens along the *z*-axis for YLP incidence.

As shown in Fig. 5a, the simulated phase profile agrees well with the theoretical one under the XLP illumination. The theoretical and simulated cross-section intensity distributions on the *xoz* plane are shown in Fig. 5b,c, respectively. Their corresponding sectional profiles along the *x*-direction are shown in Fig. 5d. The simulated full-width at half-maximum (FWHM) is about ~ 9.56 μm, which is nearly consistent with the theoretical calculation (~ 9.08 μm) by the vector angular spectrum diffraction theory^12^. For theoretical calculations, the coupling effect of adjacent supercells is omitted, and perfect phase distributions and uniform amplitude distributions are assumed. The simulated focusing efficiency is ~ 75%, which is defined as the ratio of the focused power in the region (with a radius of three times FWHM) to the transmitted power. In Fig. 5d, the theoretical and simulated radiuses of the Airy disk are 10.42 μm and 10.55 μm, respectively. The slight difference can be contributed to the fact that theoretical calculations assume perfect phase distributions and uniform amplitude. Since the designed metalens is a one-dimensional focusing lens, both theoretical and simulated radiuses are smaller than diffraction-limit (λ/2NA = 10.6 μm, NA = 0.5 is the numerical aperture) ^46,47^. However, the YLP incidence is not allowed to be transmitted by the designed metalens, as shown in Fig. 5e. The polarization extinction ratio exceeds ~ 11.6 dB. The comparative analysis (Supplementary Table S4) demonstrates that these performances of monolayer metasurfaces could approach multiple metasurfaces with maximum transmission efficiency and extinction ratio being ~ 65% and ~ 16.1 dB, respectively. These results indicate that the proposed metasurfaces enable polarization imaging with a high signal-to-noise ratio and resolution.

To fabricate the designed metasurface, the potential fabrication roadmap is shown in Fig. S4^47^. The plasma-enhanced chemical vapor deposition (PECVD)/molecular beam epitaxy (MBE) can be used to deposit a silicon layer on the cleaned MgF_2_ substrate. Then, the photoresist is spin-coated on the silicon layer and patterned by laser direct writing. Finally, the pattern can be transferred into the silicon layer by inductively coupled plasma (ICP) etching to form silicon nanofins.

## Conclusions

In summary, we propose a platform of monolayer all-dielectric metasurfaces that can simultaneously achieve polarization filtering and wavefront shaping. By localized polarization-selective constructive or destructive interference, the *x*-polarization light can be efficiently transmitted with an arbitrary wavefront while maintaining the high performance of filtering the *y*-polarization light. Under the *x*-polarization illumination, the transmission efficiency of the metasurface exceeds 75.76% at the wavelength of 10.6 μm with an extinction ratio surpassing 11.6 dB. As proof-of-concept demonstrations, a deflector and metalens have been designed and numerically simulated to exhibit high-efficiency wavefront control and high-performance polarization filtering. Such monolayer metasurfaces not only leave more space for other functions in integrated optical devices and systems but also avoids complex design and terrible near-field couplings in comparison with the multi-layer structures. It is believed that this work would have wide applications in polarization imaging, complex optical-field control, and many others.

## Supplementary information

Supplementary information.
