# Supplementary material for: Simultaneous polarization filtering and wavefront shaping enabled by localized polarization-selective interference
Source: Sci Rep. 2020 Sep 2;10:14477. doi: 10.1038/s41598-020-71508-7 (PMC7468143; doi:10.1038/s41598-020-71508-7)
Supplement: Supplementary file 1 — Supplementary information. [file 41598_2020_71508_MOESM1_ESM.pdf]

Simultaneous Polarization Filtering and Wavefront Shaping Enabled by Localized Polarization-selective Interference

Jixiang Cai[1], Fei Zhang[1], Ming Zhang[2], Yi Ou[1], and Honglin Yu[1]*

[1]Key Laboratory of Optoelectronic Technology and System, Ministry of Education, Chongqing University, Chongqing 400030, China

[2]School of Information Science and Engineering, Hebei University of Science & Technology, Shijiazhuang, 050018, China

*E-mail: hlyu@cqu.edu.cn

S1. Geometrical parameters and its effects

Table S1. Geometrical parameters of eight supercells

Geometrical parameters	Unit ID							
	1	2	3	4	5	6	7	8
L1 (μm)	1.97	2.01	2.06	2.12	2.2	2.28	2.37	2.48
L2(μm)	2.55	2.62	2.7	2.8	2.94	3.11	3.39	3.85
W1(μm)	2.94	2.75	2.57	2.39	2.2	2.02	1.86	1.69
W2(μm)	2.55	2.41	2.27	2.12	1.97	1.8	1.64	1.45
C1(μm)	0.31	0.3	0.3	0.31	0.32	0.31	0.31	0.3
C2 (μm)	0.3	0.3	0.31	0.3	0.31	0.28	0.31	0.32

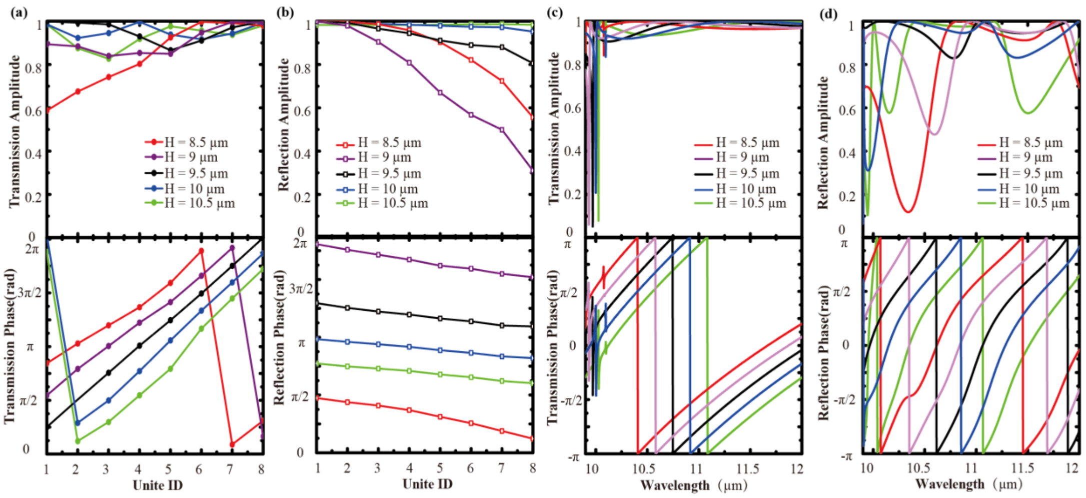

Figure S1. The effect of Si height on the (a-b) amplitude and phase of eight supercells and(c-d) the 7th supercell in Table S1 as an example. The red/blue shift can be observed in both amplitude and phase spectra as the Si height increases/reduces.

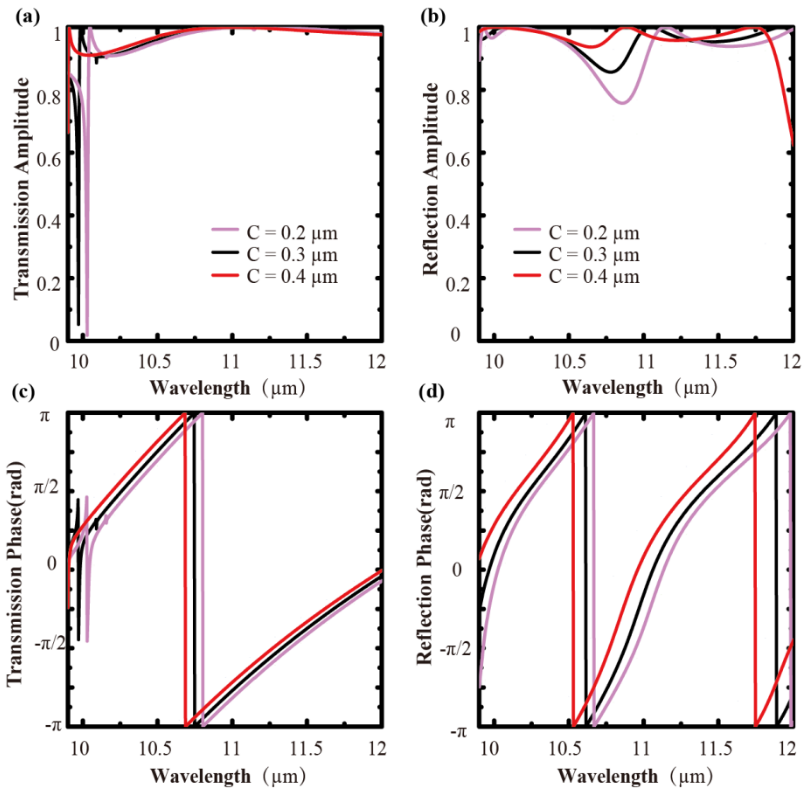

Figure S2. (a and b) The effect of the chamfer on the amplitude and (c and d) phase for the 7th supercell assuming that chamfers of C_1 and C_2 are equal to C. The slight red/blue shift for both the amplitude and phase spectra can be observed as values of charmer reduce/ increase.

S2. Performances of deflectors with different phase quantization

Table S2. Performances comparison of deflectors with different phase quantization

Phase quantization	Deflector size(μm)	Unit ID[a]	Deflection angle	Transmission efficiency	Diffraction efficiency	Extinction ratio	HPBW[b]
8-level	128	1-8	9.53 °	85.6%	92.24%	19.38 dB	4.1 °
4-level	128	1,3,5,7	19.35 °	63.51%	77.24%	18.42 dB	4.2 °
3-level	128	3,6,8	26.2 °	63.87%	95.43%	12.8 dB	4.2 °

a) Deflectors with different phase quantization are designed based on different elements with units ID in Table S1. b) HPBW means half-power beam-width.

Table S3. The effect of deflector size on HPBW for deflector with 8-level phase quantization

Deflector size (μm)	128	256	384	512	640
HPBW	4.1 °	2.1 °	1.4 °	1 °	0.8 °

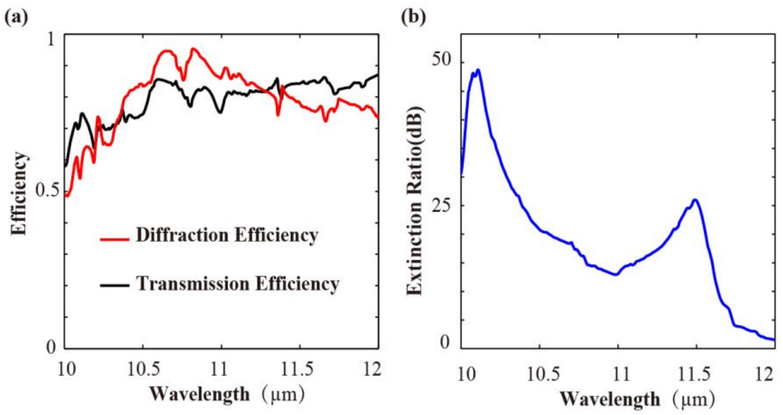

Figure S3. The (a) transmission efficiency, diffraction efficiency and (b) extinction ratio of deflector with 8-level phase quantization.

S3. Comparative analysis with presented results

Table S4. Comparative analysis with the existing results

References	wavelength	Transmission efficiency	Extinction Ratio	Material	Structure
This work	10.6μm	75%	>11.6 dB	Si	Single layer
ref[36]	632.8nm	62.25%	>16.1 dB	Au-Si	MIM
ref[37]	632.8 nm.	65%	> 10dB	Au-Si	Metal-dielectric
ref[38]	2μm	62%	-	Au-SiO_2	Cascaded multilayer
ref[39]	850nm	30%	-	Au-SiO_2	Two layers

S4. Potential fabrication roadmap

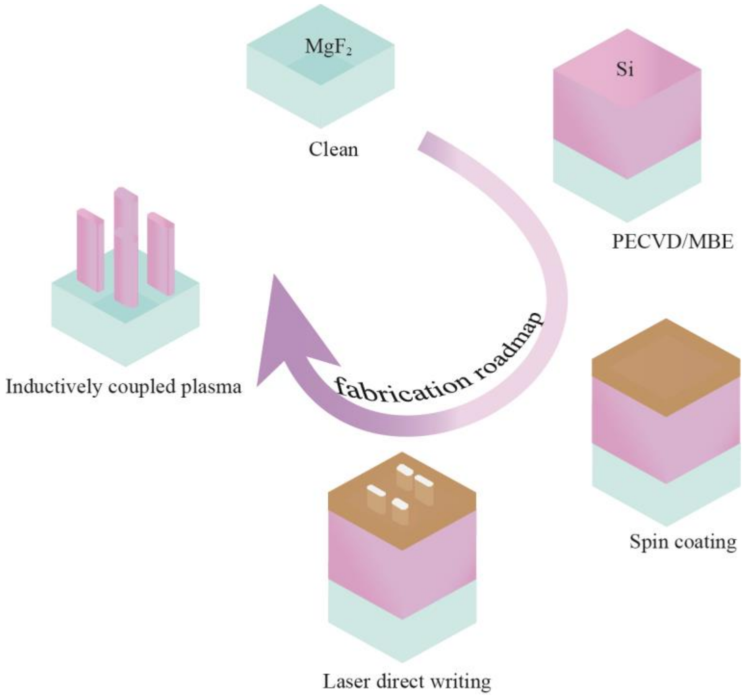

Figure S4. The potential fabrication roadmap of metalens.
